# Supplementary material for: Incorporating sparse labels into hidden Markov models using weighted likelihoods improves accuracy and interpretability in biologging studies
Source: PLoS One. 2025 Jun 18;20(6):e0325321. doi: 10.1371/journal.pone.0325321 (PMC12176159; doi:10.1371/journal.pone.0325321)
Supplement: S1 Appendix — Figures displaying results from PHMMs fit using all five values of α (PDF) [file pone.0325321.s001.pdf]

Appendix S1: additional results from case study 1

Evan Sidrow [1*], Nancy Heckman[1], Tess M. McRae[2], Beth L. Volpov[2], Andrew W. Trites[2,3], Sarah M. E. Fortune[2,4], Marie Auger-Méthé[1,2]

1 Department of Statistics, University of British Columbia, Vancouver, BC, Canada
2 Institute for the Oceans and Fisheries, University of British Columbia, Vancouver, BC, Canada
3 Department of Zoology, University of British Columbia, Vancouver, BC, Canada
4 Department of Oceanography, Dalhousie University, Halifax, NS, Canada

* evan.sidrow@stat.ubc.ca

This section recreates Figs 4 and 5 from the first case study for $\alpha = 0, 0.025, 0.049, 0.525, 1$. The results for $\alpha = 0.025$ and $\alpha = 0.525$ are very similar to those for $\alpha = 0.049$, but we include them here for completeness.

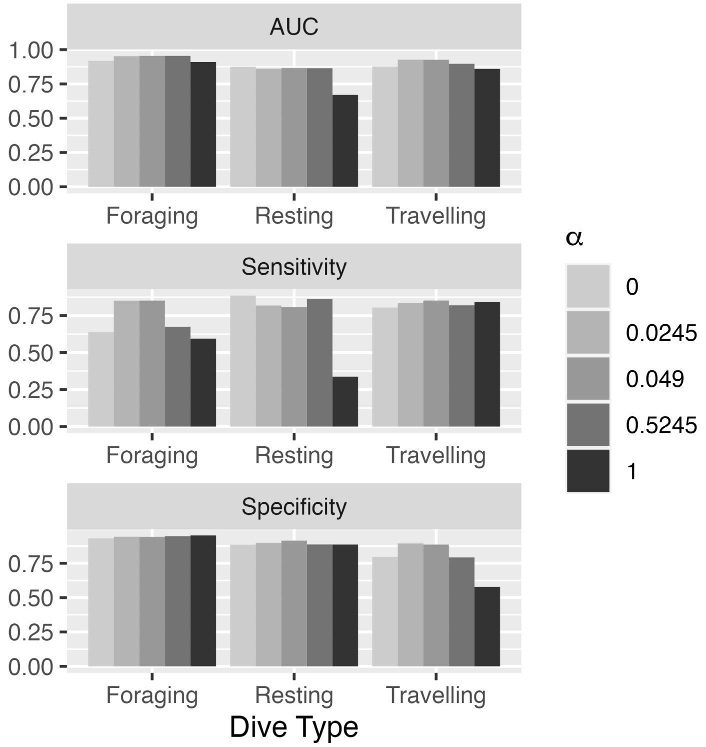

Fig S1. Sensitivity, specificity, and AUC values associated with each dive type. True values are determined via the drone-detected dive types. The PHMMs for each value of α were fit using the entire dataset with a selected sub-profile held out. Then, the dives for that sub-profile were estimated by using the forward-backward algorithm on the sub-profile with the drone-detected labels removed. We repeated this process for every sub-profile to get a full set of estimated dive types.

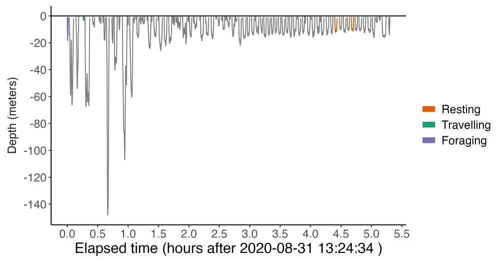

(a) Dive profile with true drone-detected labels.

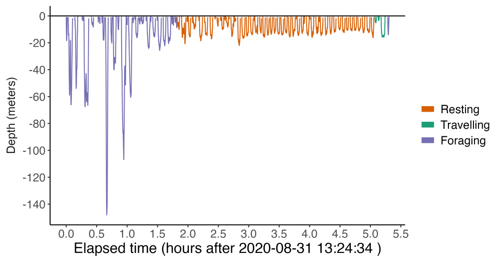

(b) Decoded dives for PHMM with $\alpha = 0.000$ (treating the unlabelled observations as missing).

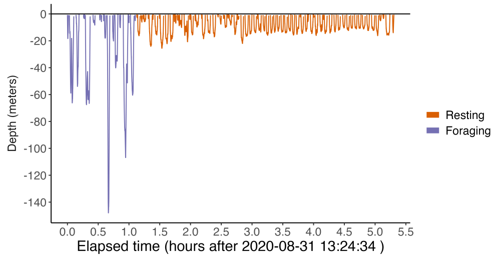

(c) Decoded dives for PHMM with $\alpha = 0.025$.

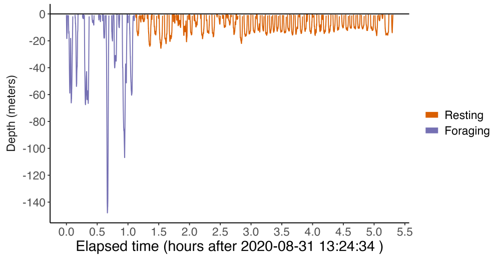

(d) Decoded dives for PHMM with $\alpha = 0.049$.

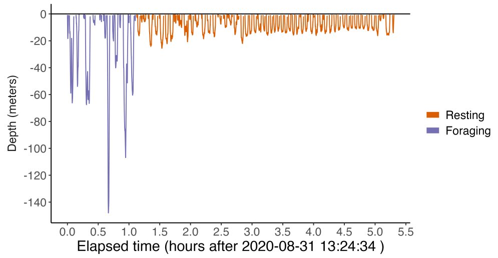

(e) Decoded dives for PHMM with $\alpha = 0.525$.

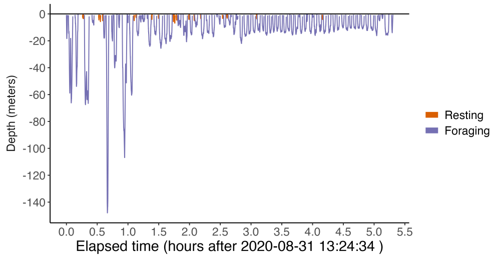

(f) Decoded dives for PHMM with $\alpha = 1.000$ (the "natural" weighting).

Fig S2. Viterbi-decoded dives of killer whale D26 (male, 10 years old) using different PHMMs. Each PHMM was fit to the dataset with the sub-profile held out. Then the Viterbi algorithm was used on the held-out dataset with its labels removed in order to test the predictive performance of each PHMM.
