# Supplementary material for: Incorporating sparse labels into hidden Markov models using weighted likelihoods improves accuracy and interpretability in biologging studies
Source: PLoS One. 2025 Jun 18;20(6):e0325321. doi: 10.1371/journal.pone.0325321 (PMC12176159; doi:10.1371/journal.pone.0325321)
Supplement: S2 Appendix — Figures displaying scatter plots of data used in the case studies and the simulation study. (PDF) [file pone.0325321.s002.pdf]

Appendix S2: figures of data distributions

Evan Sidrow [1*], Nancy Heckman[1], Tess M. McRae[2], Beth L. Volpov[2], Andrew W. Trites[2,3], Sarah M. E. Fortune[2,4], Marie Auger-Méthé[1,2]

1 Department of Statistics, University of British Columbia, Vancouver, BC, Canada
2 Institute for the Oceans and Fisheries, University of British Columbia, Vancouver, BC, Canada
3 Department of Zoology, University of British Columbia, Vancouver, BC, Canada
4 Department of Oceanography, Dalhousie University, Halifax, NS, Canada

* evan.sidrow@stat.ubc.ca

This appendix displays figures of the raw data used in case studies and simulation study of the manuscript.

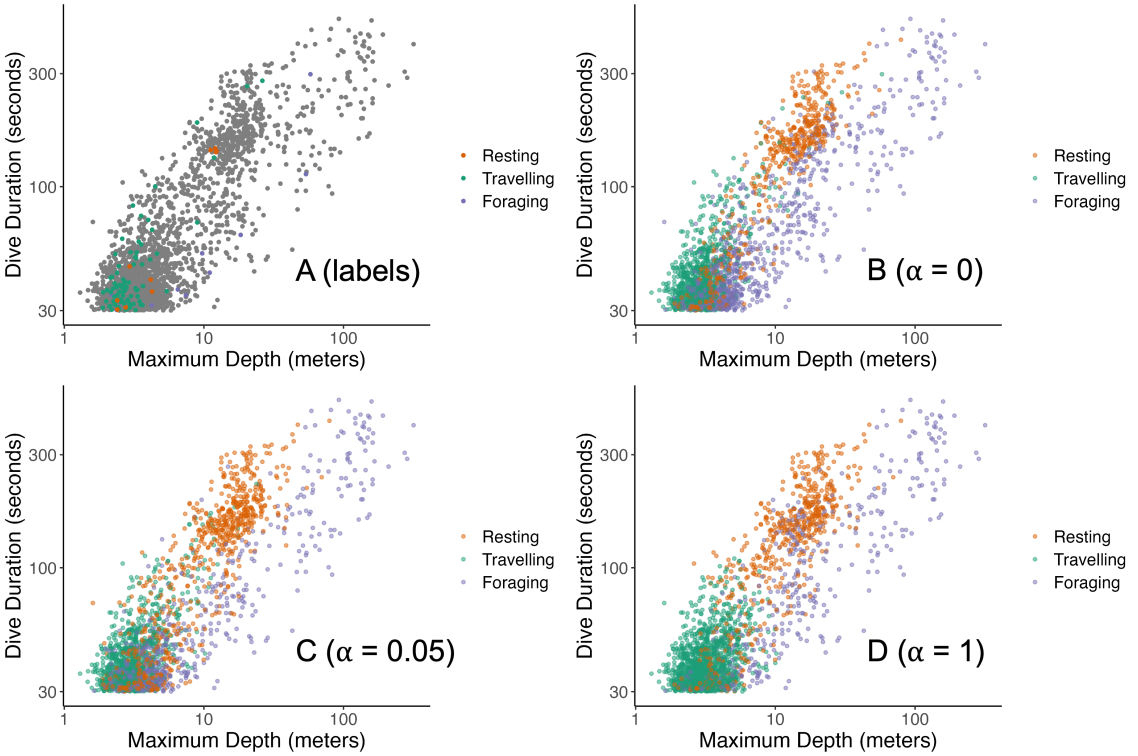

Fig S3. Scatter plot of all dive data from case study 1. Data are colour-coded either by known dive type (panel A), or by estimated dive type after using the cross-validated forward algorithm and PHMMs with $\alpha \in \{0, 0.049, 1\}$ (panels B-D). Both maximum depth and dive duration are shown on a log scale.

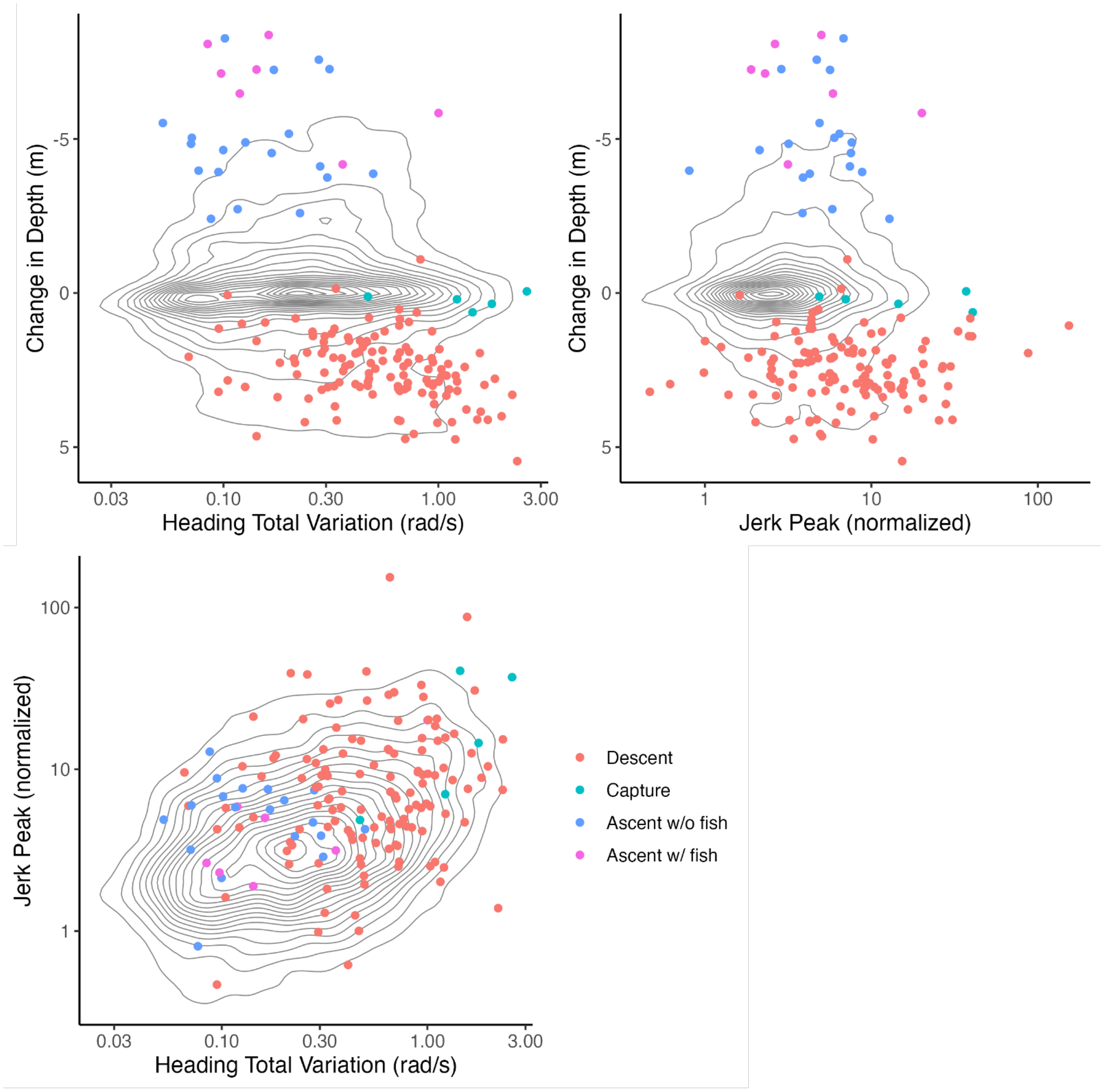

Fig S4. Scatter plots of data from labelled two-second windows. Data are colour-coded by known subdive state. A contour plot showing the estimated density of all unlabelled windows is shown in addition to the labelled points for reference. Note that heading total variation and normalized jerk peak are shown on a logarithmic scale, while the axes for change in depth are reversed (i.e., points lower on the y-axis correspond to descending).

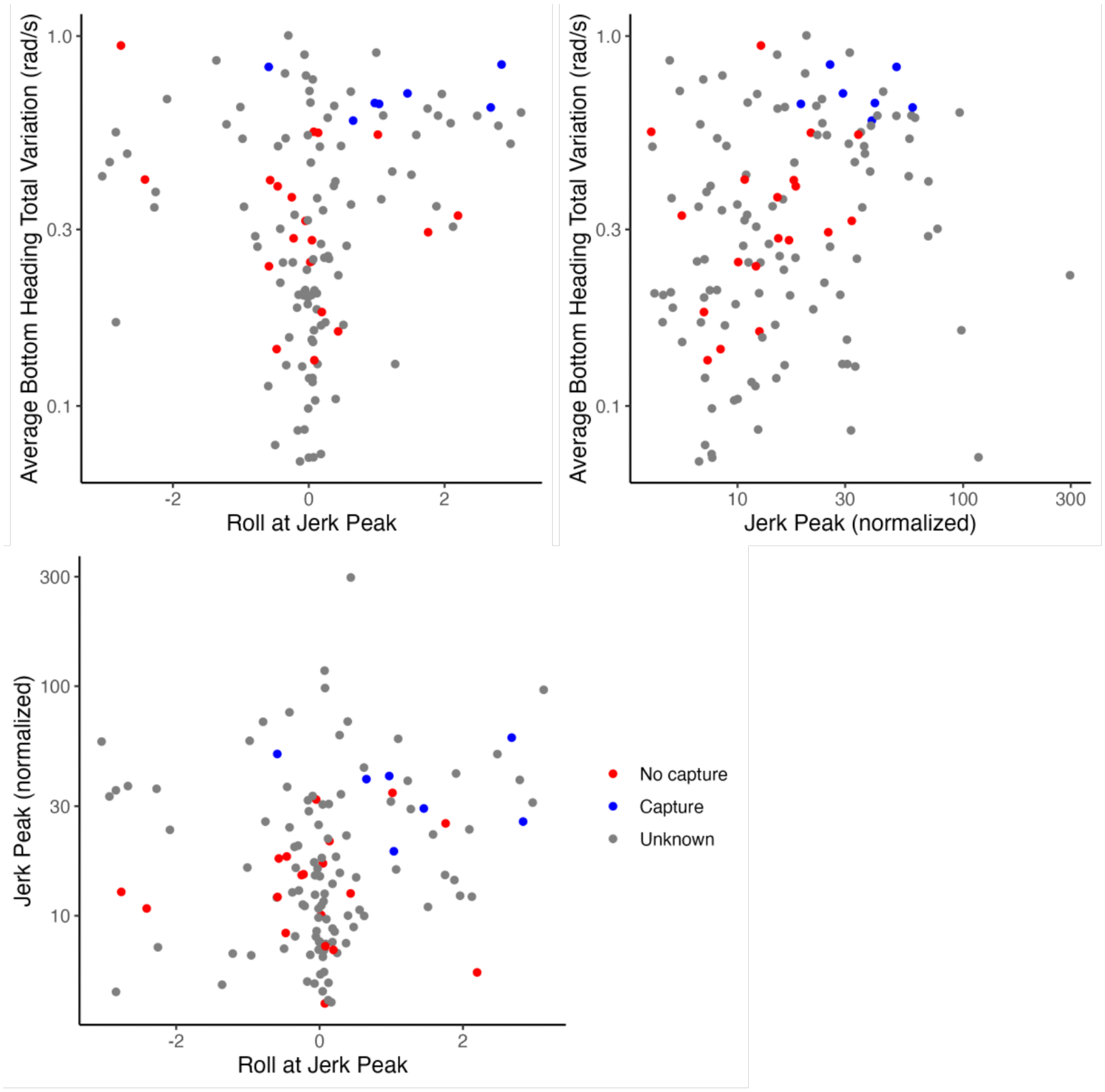

Fig S5. Scatter plots of data from labelled dives. Data are colour-coded if the corresponding dive was identified as positive or negative for successful foraging. Note that average bottom heading total variation and normalized jerk peak are shown on a logarithmic scale.

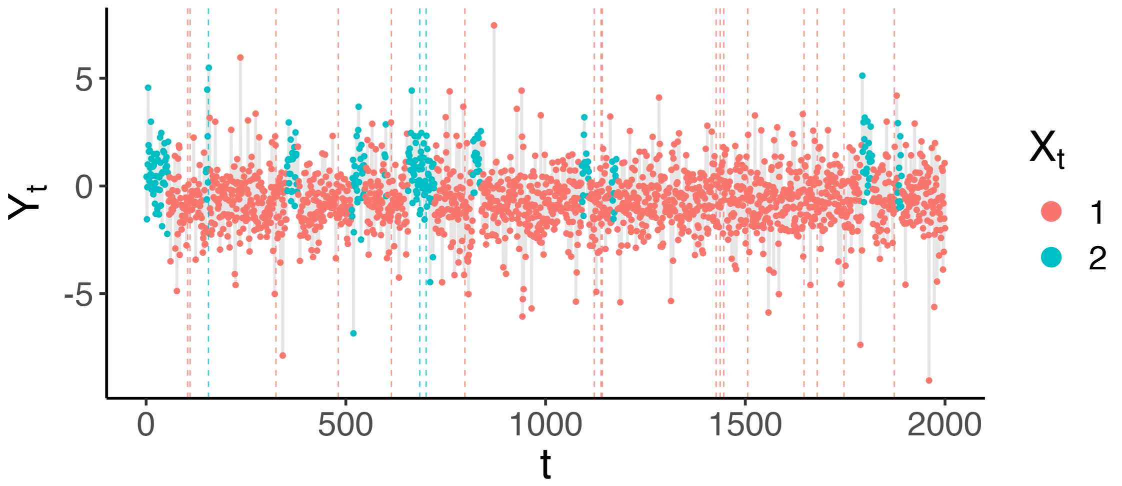

Fig S6. Simulated data set from an HMM using control simulation study settings. A total of $T = 2000$ observations were generated from an HMM using a transition matrix $\Gamma = \begin{pmatrix} 0.99 & 0.01 \\ 0.05 & 0.95 \end{pmatrix}$ and state-dependent t-distributions with $\nu = 4$ degrees of freedom whose means are $\kappa = 1$ standard deviations apart. A total proportion of $\ell = 0.01$ of all observations are labelled, as indicated by vertical dashed lines.
