# Supplementary material for: Incorporating sparse labels into hidden Markov models using weighted likelihoods improves accuracy and interpretability in biologging studies
Source: PLoS One. 2025 Jun 18;20(6):e0325321. doi: 10.1371/journal.pone.0325321 (PMC12176159; doi:10.1371/journal.pone.0325321)
Supplement: Attachment — Submitted filename: PHMM_PLOS_reviewer_responses.rtf [file pone.0325321.s003.rtf]

Dear Dr. Sidrow,

Thank you for submitting your manuscript to PLOS ONE. After careful consideration, we feel that it has merit but does not fully meet PLOS ONE’s publication criteria as it currently stands. Therefore, we invite you to submit a revised version of the manuscript that addresses the points raised during the review process.

Please submit your revised manuscript by Jan 29 2025 11:59PM. If you will need more time than this to complete your revisions, please reply to this message or contact the journal office at plosone@plos.org. When you're ready to submit your revision, log on to https://www.editorialmanager.com/pone/ and select the 'Submissions Needing Revision' folder to locate your manuscript file.

Please include the following items when submitting your revised manuscript:
	•	A rebuttal letter that responds to each point raised by the academic editor and reviewer(s). You should upload this letter as a separate file labeled 'Response to Reviewers'.
	•	A marked-up copy of your manuscript that highlights changes made to the original version. You should upload this as a separate file labeled 'Revised Manuscript with Track Changes'.
	•	An unmarked version of your revised paper without tracked changes. You should upload this as a separate file labeled 'Manuscript'.
If you would like to make changes to your financial disclosure, please include your updated statement in your cover letter. Guidelines for resubmitting your figure files are available below the reviewer comments at the end of this letter.  If applicable, we recommend that you deposit your laboratory protocols in protocols.io to enhance the reproducibility of your results. Protocols.io assigns your protocol its own identifier (DOI) so that it can be cited independently in the future. For instructions see: https://journals.plos.org/plosone/s/submission-guidelines#loc-laboratory-protocols. Additionally, PLOS ONE offers an option for publishing peer-reviewed Lab Protocol articles, which describe protocols hosted on protocols.io. Read more information on sharing protocols at https://plos.org/protocols?utm_medium=editorial-email&utm_source=authorletters&utm_campaign=protocols.  We look forward to receiving your revised manuscript.
Kind regards,
Vitor Hugo Rodrigues Paiva, Ph.D. Academic Editor PLOS ONE
Journal Requirements:
When submitting your revision, we need you to address these additional requirements.
1. Please ensure that your manuscript meets PLOS ONE's style requirements, including those for file naming. The PLOS ONE style templates can be found at  https://journals.plos.org/plosone/s/file?id=wjVg/PLOSOne_formatting_sample_main_body.pdf and  https://journals.plos.org/plosone/s/file?id=ba62/PLOSOne_formatting_sample_title_authors_affiliations.pdf

We have read the style templates above to ensure that our manuscript meets PLOS ONE’s style requirements.

2. Please note that PLOS ONE has specific guidelines on code sharing for submissions in which author-generated code underpins the findings in the manuscript. In these cases, all author-generated code must be made available without restrictions upon publication of the work. Please review our guidelines at https://journals.plos.org/plosone/s/materials-and-software-sharing#loc-sharing-code and ensure that your code is shared in a way that follows best practice and facilitates reproducibility and reuse.

All of our code is available at https://github.com/EvanSidrow/PHMM

3. In your Methods section, please provide additional information regarding the permits you obtained for the work. Please ensure you have included the full name of the authority that approved the field site access and, if no permits were required, a brief statement explaining why. 

We have added the following statement at the beginning of our “Case studies” section to provide additional information about our permits.

The data for these case studies were collected with written approval under the University of British Columbia Animal Care Permit no. A19-0053, Fisheries and Oceans Canada Marine Mammal Scientific License for Whale Research no. XMMS 6 2020, and United States Department of Commerce, NOAA, National Marine Fisheries Service Permit No. 23220. Collection took place in August and September 2020 off the coast of British Columbia in Queen Charlotte Sound, Queen Charlotte Strait, Johnstone Strait, and Juan de Fuca Strait.
 
4. Please include your full ethics statement in the ‘Methods’ section of your manuscript file. In your statement, please include the full name of the IRB or ethics committee who approved or waived your study, as well as whether or not you obtained informed written or verbal consent. If consent was waived for your study, please include this information in your statement as well.

We believe that our statement above at the beginning of our “Case studies” addresses this point, but please let us know if anything else is needed.
 
5. We note you have included a table to which you do not refer in the text of your manuscript. Please ensure that you refer to Table 2 in your text; if accepted, production will need this reference to link the reader to the Table.

To the best of our knowledge we have no tables in our manuscript. We had referred to Table 2 of McRae et al. (2024), but we did not explicitly show this table. We have removed the reference to Table 2, and instead simply reference McRae et al. (2024) to avoid confusion.   [Note: HTML markup is below. Please do not edit.]  Reviewers' comments:  Reviewer's Responses to Questions
Comments to the Author  1. Is the manuscript technically sound, and do the data support the conclusions?  The manuscript must describe a technically sound piece of scientific research with data that supports the conclusions. Experiments must have been conducted rigorously, with appropriate controls, replication, and sample sizes. The conclusions must be drawn appropriately based on the data presented.
Reviewer #1: Partly
Reviewer #2: Yes

2. Has the statistical analysis been performed appropriately and rigorously?
Reviewer #1: Yes
Reviewer #2: Yes

3. Have the authors made all data underlying the findings in their manuscript fully available?  The PLOS Data policy requires authors to make all data underlying the findings described in their manuscript fully available without restriction, with rare exception (please refer to the Data Availability Statement in the manuscript PDF file). The data should be provided as part of the manuscript or its supporting information, or deposited to a public repository. For example, in addition to summary statistics, the data points behind means, medians and variance measures should be available. If there are restrictions on publicly sharing data—e.g. participant privacy or use of data from a third party—those must be specified.
Reviewer #1: Yes
Reviewer #2: Yes

4. Is the manuscript presented in an intelligible fashion and written in standard English?  PLOS ONE does not copyedit accepted manuscripts, so the language in submitted articles must be clear, correct, and unambiguous. Any typographical or grammatical errors should be corrected at revision, so please note any specific errors here.
Reviewer #1: Yes
Reviewer #2: Yes

5. Review Comments to the Author  Please use the space provided to explain your answers to the questions above. You may also include additional comments for the author, including concerns about dual publication, research ethics, or publication ethics. (Please upload your review as an attachment if it exceeds 20,000 characters)
Reviewer #1: This study introduces a weighted likelihood approach for hidden Markov models (HMMs) to address the challenge of sparse labeling in ecological time series data. Through case studies on killer whale foraging behavior, the approach demonstrates notable improvements in accuracy and interpretability compared to existing methods. Overall, the manuscript is well-organized and effectively presented. However, several enhancements could further strengthen its completeness and rigor.

Thank you for the kind words and constructive comments. We believe the changes we made in response to the comments further strengthen the manuscript.

 Data Visualization: It would be highly beneficial to include plots that showcase the actual distribution of the data points. For Case Study 1, a 2D scatter plot of maximum depth ( m ) against dive duration ( d ), color-coded by dive types, would provide an intuitive visualization of the data. Similarly, for Case Study 2, a 3D scatter plot of depth change (d) , heading variation ( h ), and jerk peak ( j ), color-coded by labels, would help illustrate the feature space and label distribution.

We agree that scatter plots improve the interpretability of our case studies. We have added scatter plots that show the distribution of the data points for case study 1 (Fig S3) and case study 2 (Fig S4 and Fig S5). We have also plotted an example data set from our new simulation study (Fig S6). All figures are in Appendix S2.
 Comparison with Single-Frame Methods: Please perform a side-by-side comparison of the proposed HMM-based methods (with varying \alpha ) against single-frame methods, such as random forest, SVM, etc. Intuitively, the temporal information utilized by multi-frame HMMs should lead to better performance than single-frame methods, while the proposed weighted likelihood approach is expected to outperform standard HMMs due to the inclusion of label weighting. Quantifying these performance improvements in a clear and systematic manner would provide strong support for the method’s efficacy.
 Thank you for this suggestion. We have added three single-frame methods to case studies 1 and 2: logistic regression, random forests, and SVMs (See line number 339-342). The PHMMs outperform the single frame methods in case study 1, but are matched by single frame methods in case study 2. We believe this is because the labelled foraging dives are well-separated based on average bottom heading variation and jerk peak (see our new Fig S5). Nonetheless, we believe that our method is still useful for case study 2 because it is as accurate as the single frame methods but simultaneously categorizes dive phases as well as successful foraging dives. It also infers the underlying generative process for the killer whale data.
 Equation and Figure Clarifications: In Equation (2), the parameter \delta should be explicitly defined for clarity. Additionally, in Figure 2, it is difficult to distinguish between rows A, B, and C. Enhancing the figure with more distinct visual markers or annotations would improve its readability and interpretation.

We have added an Equation to define delta more clearly (now Equation (1)), and have added additional wording prior to Equation (2) (now Equation (3)) to clarify \delta (lines 84-86). We have also adjusted Figure 2 to distinguish more clearly between rows A, B, and C.

Reviewer #2: This paper provides a detailed description of a novel statistical method for incorporating partially labelled data in hidden Markov models to estimate more detailed state processes in animal movement studies. This paper functions both as a methodology paper, and does a good job of describing the statistical theory and methods, as well as providing novel ecological results in the case studies about killer whale foraging behavior. 

Thank you for the kind words and constructive comments. The changes we made in response to them have improved the manuscript.

While I appreciate the ecological relevance of the killer whale case studies, from a methodological point of view I would have loved to see an additional case study with a comparison to a case where there is more labelled data, or the data labelling comes from multiple processes. I think this would help the reader better understand when they should choose to use this method. 

While a new case study would add to the manuscript, we believe that a simulation study would better demonstrate when the method is most useful in a controlled setting, which we understood as the main comment here. As such, we have added a simulation study after the case studies to investigate the effect of label density on the ideal value of alpha. The simulation study also investigates the effects of model misspecification, state separation, data set size, and class imbalance. We determined that all of these factors can affect the ideal value of alpha. In summary, we conclude that our method is useful in difficult modelling scenarios (fewer labels, more model misspecification, less state separation, etc.). However, it is difficult to know in practice how the data affects the ideal value of alpha, so we recommend using our approach with cross-validation to find the ideal value of alpha. Also note that our method is a generalization of the standard HMM approach. (lines 563-634).

Additionally, I was confused about the way the cross-validation procedure worked, especially in terms of using individual killer whale data but in different folds. I suggest that the authors rewrite these sections to better clarify this procedure, as well as to perhaps include a conceptual diagram. Overall, I believe this paper is a valuable contribution to the field and should be published.

We have re-worded the cross-validation procedure  (lines 207-231) and added a conceptual diagram (Fig 3).
 Minor comments: Line 15: re-word “what animals are doing”, perhaps: “fine-scale animal behavior” 

We have made the suggested change (line 26).

Line 18: Can you be more specific about what “various settings” means here?

We have changed the wording in this sentence to clarify (line 29).
 Line 45-46: These paragraphs do not feel well connected, perhaps add a transition sentence between the two 

We agree that the paragraph is out of place. As such, we have moved the second paragraph earlier in the introduction to make the flow of the introduction better (lines 15-25). 
 Line 48: I believe “Northern” and “Southern” are traditionally capitalized in the context of killer whales

We have double checked the literature and have found mixed results: some papers use upper-case and some use lower-case. However, we have found that “northern” and “southern” generally tend to be lower-case. Some examples include:
https://www.sararegistry.gc.ca/document/doc1341a/p1_e.cfm
https://onlinelibrary.wiley.com/doi/epdf/10.1111/gcb.17490
https://cdnsciencepub.com/doi/10.1139/cjfas-2020-0445

 Lines 68 - 74: I think it would be useful somewhere in here to specify that states are discrete but that observations are usually continuous 

We have made this change in the manuscript (lines 72-74).

 Lines 67 - 86: I’m not sure you need the full description of HMMs here, as they are relatively commonly used these days. Perhaps this section can be shortened.

While we agree that HMMs are relatively common and that most readers can skip this subsection, we had trouble balancing this request with one from another reviewer to expand on the section. The section also introduces notation that is used later in the manuscript.
 Line 129: Replace “throws out” with “removes” 

We have made this change in the manuscript (line 135).
 Line 279: I noticed that in both case studies you match the state structure with the number of states in your labels (3 dive types, 3 states) what would happen if these did not match? 

This is an important observation, and this phenomenon can cause complications in the model. In fact, in the second case study we do not match the state structure with the number of distinct label types. If there are more states in the HMM than there are distinct label types, then it is not advisable to set $\alpha = 0$, since then there would be no information to estimate any unlabelled hidden states. We mentioned this problem briefly in the second case study, but we have moved the explanation of the issue up to the “model” section because it is an important consideration for all PHMMs. In addition, if a single label could correspond to multiple components (e.g. there are two kinds of foraging but a foraging label cannot distinguish between the two), then we recommend parameterizing g^{(i)}. We have clarified this on lines 126 as well as lines 213-216.
 Lines 368-370: I understand the biological reason for limiting the data to dives > 30 m, but was there a statistical reason as well? How might this apply to other taxa?

From a statistical standpoint, the HMM from the second case study is likely a bad model for dives < 30m because heading total variation, jerk peak, etc. is likely distributed differently for shallow dives compared to deep foraging dives. We have added this information to case study 2 in lines 412-414. In addition, we believe that our modelling approach can work for other taxa, but dividing up foraging and non-foraging dives should be made on a case-by-case basis. We have added this to the discussion section of the manuscript in lines 663-666.

 Figure 3: I think if you remove the gray background to these plots it will help make the gray bars more distinguishable

Thank you for the suggestion- we have made the changes in the manuscript (now Figure 4).


6. PLOS authors have the option to publish the peer review history of their article (what does this mean?). If published, this will include your full peer review and any attached files.   If you choose “no”, your identity will remain anonymous but your review may still be made public.   Do you want your identity to be public for this peer review? For information about this choice, including consent withdrawal, please see our Privacy Policy.
Reviewer #1: No
Reviewer #2: No
